# Controllability of Vortex Domain Structure in Ferroelectric Nanodot: Fruitful Domain Patterns and Transformation Paths

**DOI:** 10.1038/srep03946

**Published:** 2014-02-04

**Authors:** C. M. Wu, W. J. Chen, Yue Zheng, D. C. Ma, B. Wang, J. Y. Liu, C. H. Woo

**Affiliations:** 1 State Key Laboratory of Optoelectronic Materials and Technologies, Micro&Nano Physics and Mechanics Research Laboratory, School of Physics and Engineering, Sun Yat-sen University, Guangzhou 510275, China; 2 Sino-French Institute of Nuclear Engineering and Technology, Zhuhai Campus, Sun Yat-sen University, Zhuhai 519082, China; 3 Department of Physics and Materials Science, City University of Hong Kong, Hong Kong SAR, China

## Abstract

Ferroelectric vortex domain structure which exists in low-dimensional ferroelectrics is being intensively researched for future applications in functional nanodevices. Here we demonstrate that adjusting surface charge screening in combination with temperature can provide an efficient way to gain control of vortex domain structure in ferroelectric nanodot. Systematical simulating experiments have been conducted to reveal the stability and evolution mechanisms of domain structure in ferroelectric nanodot under various conditions, including processes of cooling-down/heating-up under different surface charge screening conditions, and increasing/decreasing surface charge screening at different temperatures. Fruitful phase diagrams as functions of surface screening and temperature are presented, together with evolution paths of various domain patterns. Calculations discover up to 25 different kinds of domain patterns and 22 typical evolution paths of phase transitions. The fruitful controllability of vortex domain structure by surface charge screening in combination with temperature should shed light on prospective nanodevice applications of low-dimensional ferroelectric nanostructures.

Ferroelectric materials have received considerable attentions from both academics and industries owing to their broad applications in multifunctional electric devices, such as memories, actuators and sensors, etc^1^,^2^,^3^,^4^,^5^. In the pursuit of device miniaturization and high performance, more and more ferroelectric nanostructures are being exploited as basic functional device components in recent years. However, properties of ferroelectric nanostructures are quite different from those of their bulk counterparts due to the complicated coupling effects of bulk ferroelectricity with surface or interface^6^,^7^,^8^,^9^,^10^. Due to the large surface to volume ratio of the nanostructures, their properties are expected to be sensitive to the system's geometry, size, and the surrounding environment. Owing to this fact, widespread research has been driven onto ferroelectric nanostructures and the exploration of their novel performance. At present, increasing amount of high quality ferroelectric nanostructures are being successfully fabricated in experiments and have received intensive characterizations and analyses^11^,^12^,^13^,^14^,^15^. Meanwhile, development of various theoretical methods have been demonstrated to be effective in analyzing and predicting the properties of ferroelectric nanostructures, including first-principle calculations, atomistic level simulations and thermodynamic approaches, etc^7^,^16^,^17^,^18^,^19^,^20^,^21^.

A common feature for ferroelectrics is the formation of domain structure as the material is cooled down through Curie temperature, due to the coexistence of different energetically equivalent polar domains. In general, stability and features of domain structure, e.g., domain configuration, domain size and domain wall thickness, depend on the delicate balance of the electromechanical energies that are sensitive to the boundary conditions, which is especially true for ferroelectric nanostructures. Particularly, recent researches have shown that low-dimensional ferroelectrics can form a special type of domain structure with closure polar domains, namely vortex domain structure (VDS)^1^,^13^,^15^,^22^,^23^,^24^,^25^,^26^,^27^,^28^,^29^. Due to its distinct features from conventional domain structures, VDS in ferroelectrics indicates novel potential applications, and arouses huge interests to the field of domain and domain wall engineering. One of the well known potential applications is to store data by ferroelectric vortices, which is promising to develop high-density memory^1^, due to the small size and weak electrostatic interaction between vortices. Moreover, ferroelectric nanowire with ferroelectric-ferrotoroidic transition has been predicted to show giant reverse piezoelectric response, revealing a new potential application of VDS in sensor devices^30^.

It has been demonstrated that the domain structure in ferroelectrics could be dramatically changed by electric field, mechanical field and temperature, etc., which forms the base of domain and domain wall applications^6^,^7^,^19^,^31^,^32^,^33^,^34^,^35^,^36^. It would be much instructive for the application of VDS in ferroelectrics if the mechanisms of its formation and transformation are understood. In literature, VDS in low-dimensional ferroelectrics has been detected by high resolution electron microscopy and piezoresponse force microscopy (PFM)^13^,^14^,^37^,^38^. Theoretical works are now focusing on controlling VDS using various electric or mechanical means^19^,^31^,^34^,^35^,^36^,^39^,^40^,^41^. For example, it was found that the vortex orientation could be efficiently controlled by placing charged tips near the ferroelectric nanodot^35^. Moreover, Chen et al. investigated the possibility of controlling VDS by applying mechanical loads and demonstrated novel VDS transformations induced by mechanical loads^34^,^36^.

It is worth noting that surface charge screening should be also effective to influence VDS, considering the sensitivity of its formation on the electric boundary condition^31^. In general, controllable screening of surface polarization charges in ferroelectric nanostructures can be achieved in two approaches. One is using electrode to control the extent of charge screening, which is much associated with the intrinsic electrode properties and the contact distance between the electrode and the ferroelectrics. The other way is to use charged gas molecules or other charged particles in the environment or on the surface to screen electric field of the ferroelectrics. Here the chemical equilibrium of gas absorption can be controlled conveniently by gas pressure. As surface charge screening is a general feature of materials with surface/interface, studying its influence is of practical significance. Nevertheless, up to now there are very few investigations on the influence of surface charge screening on the VDS in low-dimensional ferroelectrics. Furthermore, reported works mainly focused on the formation mechanism of VDS rather than its evolution behavior when the screening condition changes. More systematical research on the controlling VDS by surface charge screening is needed.

In this work, we perform phase-field simulations on ferroelectric nanodots under variable surface charge screening conditions. Based on the important effect of surface charge screening on the domain structure and the exigent need of obtaining transformation paths of domain structure, simulating experiments of nanodots under processes of increasing/decreasing charge screening are conducted. In addition, owing to the practical application of nanodevices in complicated environment, the effects of temperature variation (i.e., cooling-down/heating-up processes) on the domain structure of nanodots are also investigated systematically. Fruitful domain patterns, transformations and phase diagrams are revealed and summarized.

## Results

**Figure 1:**
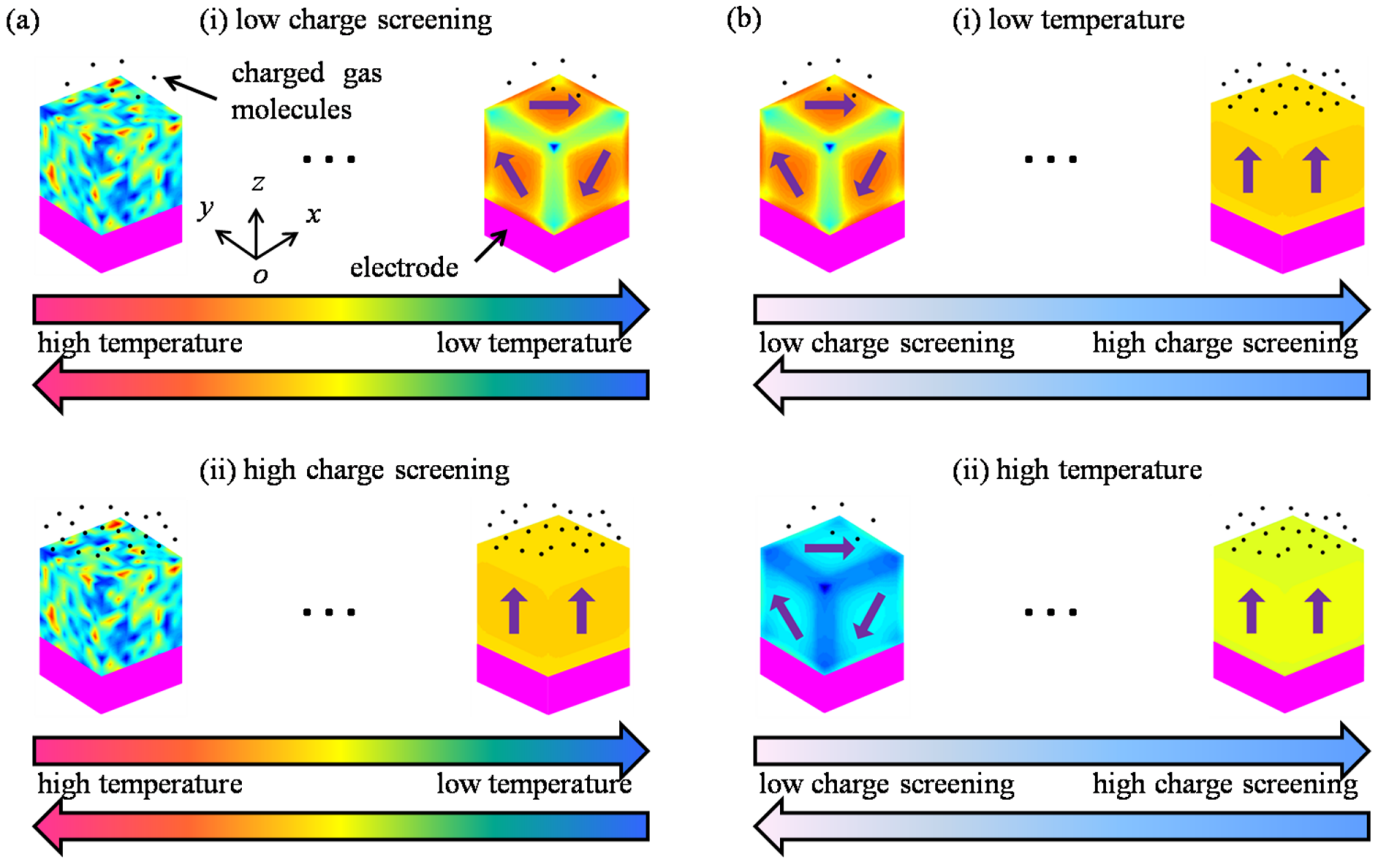
Schematics of simulating experiments on a ferroelectric nanodot and possible evolution paths of its domain structure. (a) Processes of cooling-down and heating-up under different surface charge screening conditions, and (b) processes of increasing and decreasing surface charge screening at different temperatures.

Our investigation is organized into four main sections according to the important simulating experiments as schematically illustrated in Figure 1. First, we study the evolution of the domain structure in the ferroelectric nanodot in cooling-down process through paraelectric phase under different surface charge screening conditions (Figure 1a). The domain patterns at lower temperature are evolved from those obtained at higher temperature. To see whether there is hysteresis in the evolution of domain structure, ferroelectric nanodot in heating-up process is also studied (Figure 1a), with the domain patterns at higher temperature evolved from those at lower temperature. In the last two sections, we present the evolution picture of the domain structure in the nanodot under variable charge screening conditions at fixed temperatures, i.e., increasing charge screening from open-circuit to short-circuit boundary condition and the reverse process of decreasing charge screening (Figure 1b). The domain patterns under the current charge screening condition are evolved from those obtained under the previous charge screening condition. These four simulating experiments present us a comprehensive picture of surface charge screening effects on the domain structure in ferroelectric nanodot. In this paper, we take the free-standing BaTiO_3_ nanodot as a model system to demonstrate the effect of charge screening and temperature on the VDS of the ferroelectric nanostructures. Some necessary information of bulk BaTiO_3_, i.e., phases, their temperature ranges, and polarization at selected temperatures, has been listed in Supplementary Table S1 on line to have a better understanding on the behavior of BaTiO_3_ nanodot revealed in the following.

The ferroelectric nanodot is supposed to be under charge screening at the top and bottom surfaces, i.e., axial screening along the *z* direction. When the nanodot is under ideal open-circuit boundary condition, the depolarization field would be the maximum owing to no polarization charge being compensated. As surface charge screening increases, the depolarization field would decrease and achieve the minimum under ideal short-circuit boundary condition. Basing on this fact, when the nanodot is under charge screening, we can approximate the extent of charge screening by introducing screening factors *β_i_* (*i* = 1, 2, 3), and the depolarization field in the nanodot is calculated by 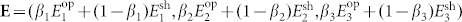, where 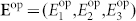 and 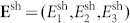 are the depolarization fields under ideal open-circuit condition and ideal short-circuit condition, respectively. As *β_i_* vary from zero to one, the charge screening condition of the nanodot changes from the ideal short-circuit boundary condition (*β_i_* = 0) to the ideal open-circuit boundary condition (*β_i_* = 1) accordingly. In our investigation, the charge screening is at the top and bottom surfaces, i.e., *β*_1_ = *β*_2_ = 1, 0 ≤ *β*_3_ ≤ 1. To clearly characterize each domain pattern, we calculate its average polarization vector 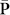 and the toroidal moment 
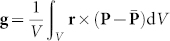
27, in which **r** is the position vector, *V* is the volume of the nanodot, and **P** is the spontaneous polarization vector. The average magnitude of polarization at all the sites 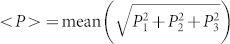 is also introduced to denote the paraelectric-ferroelectric phase transition.

**Figure 2:**
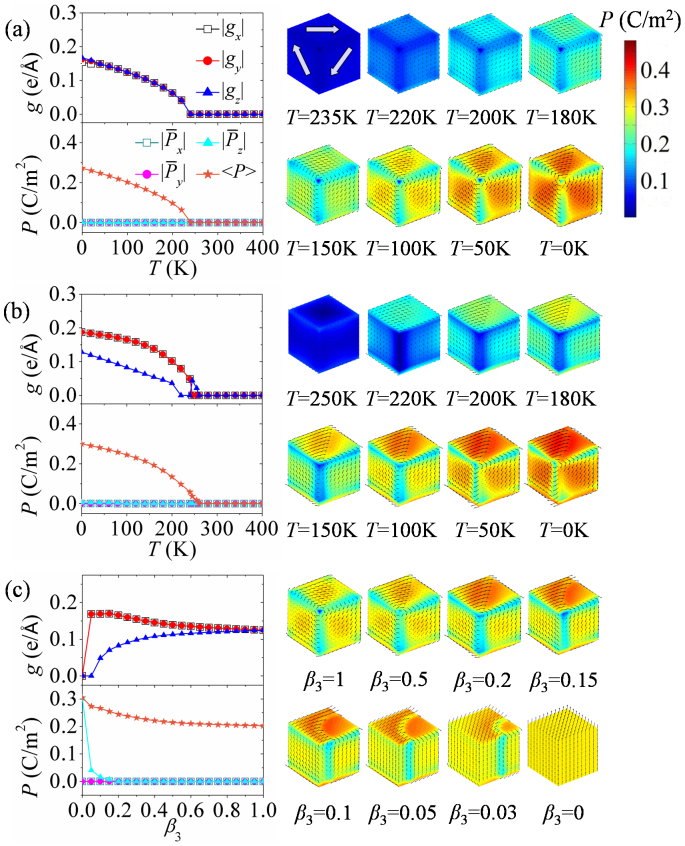
Simulated results of toroidal moments, polarizations and equilibrium domain patterns of a nanodot under charge screening condition. (a) *β*_3_ = 1 (open-circuit) and (b) *β*_3_ = 0.2 during cooling-down processes. (c) Those in increasing surface charge screening process at temperature *T* = 100 K.

First of all, we would like to see whether surface charge screening would affect the domain structure obviously. Figures 2a and 2b depict the simulation results of two cooling-down processes of nanodots under different surface charge screening conditions, i.e., ideal open-circuit condition (*β*_3_ = 1) and near short-circuit condition (*β*_3_ = 0.2), respectively. For each cooling-down process, a random perturbation of polarization field is introduced to initiate the polarization evolution at high temperature. Once a stable domain pattern is formed below paraelectric-ferroelectric transition temperature, it is used as the initial domain pattern for the next temperature step. When cooled down under open-circuit boundary condition (Figure 2a), the nanodot begins to form a domain pattern at *T* = 235 K, accompanied by nonzero toroidal moment **g** and average polarization magnitude <*P*>, but zero net polarization 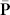. The much lower transition temperature than that of bulk BaTiO_3_ (~398 K) indicates a strong size effect of the nanodot. For this domain pattern, the three components of the toroidal moment are almost equal with each other, i.e., |*g_x_*| ≈ |*g_y_*| ≈ |*g_z_*|. From the morphologies of equilibrium domain pattern in Figure 2a, we can see that this domain pattern actually has a single vortex with its toroidal axis along the <111> direction, in consistent with the prediction of a rhombohedral vortex domain pattern by previous works^27^,^33^. As the temperature further decreases, this rhombohedral vortex domain pattern keeps stable with its toroidal moment increasing gradually. One should note that unlike the bulk BaTiO_3_ with tetragonal, orthorhombic and rhombohedral ferroelectric phases, tetragonal and orthorhombic vortex domain patterns are suppressed in the nanodot under ideal open-circuit condition. Nevertheless, this may become different in case of charge screening conditions. Indeed, when the nanodot is cooled down under screening condition *β*_3_ = 0.2, i.e., with most top and bottom surface charges being compensated, a vortex domain pattern with vortex axis along <100> direction (|*g_x,y_*| = 0, |*g_z_*| ≠ 0) forms between 245 K and 255 K as seen in Figure 2b. As the temperature continues decreasing, a perfect orthorhombic vortex domain pattern (|*g_x_*| = |*g_y_*|, |*g_z_*| = 0) appears and keeps stable until *T* reaches 215 K. Then the nanodot changes into orthorhombic-like vortex domain pattern, whose *z*-component of toroidal moment is less than the other two components. Therefore, the electric boundary condition could largely affect the stability of VDS in the ferroelectric nanodot. Here we would like to point out that elastic energy and gradient energy also play important roles in affecting the domain stability besides electric energy. Actually, tetragonal, orthorhombic and rhombohedral domain patterns would emerge under ideal open-circuit condition if the elastic energy is artificially switched off. Meanwhile, tetragonal and rhombohedral phases would appear under ideal open-circuit condition in cooling-down process when the gradient energy coefficients are set as isotropy, e.g., *G*_11_ = 3.46 × 10^−10^ Jm^3^C^−2^, G_12_ = 0 Jm^3^C^−2^, G_44_ = 1.73 × 10^−10^ Jm^3^C^−2^. These results together indicate that the domain stability of ferroelectric nanodot is a result of complicated factors.

The above calculation presents the effect of temperature on domain structure in a nanodot under fixed surface charge screening. To know how the domain structure would evolve when the surface charge screening changes, we further simulate a nanodot in process of increasing surface charge screening from ideal open-circuit condition to ideal short-circuit condition at *T* = 100 K. From Figure 2c we can see that the rhombohedral vortex domain pattern under open-circuit boundary condition would initially rotate its axis from <111> direction towards *x*-*y* plane with the increasing of surface charge screening, which indicates the appearance of orthorhombic-like vortex domain pattern (|*g_x_*| ≈ |*g_y_*| > |*g_z_*|). In the process of *β*_3_ decrease from 0.16 to 0, the average polarization component 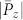 rises up gradually, which indicates that the domain pattern has both toroidal and polar features. The net polarization reaches a maximum under the ideal short-circuit boundary condition, where a single polar domain pattern is formed. From the results of Figure 2, it can be clearly seen that the characteristic of VDS formation in the nanodot during cooling-down process indeed significantly depends on surface charge screening (Figures 2a and 2b), and VDS transformations can be induced by adjusting surface charge screening on the nanodot (Figure 2c).

### Nanodot in cooling-down process under various charge screening conditions

**Figure 3:**
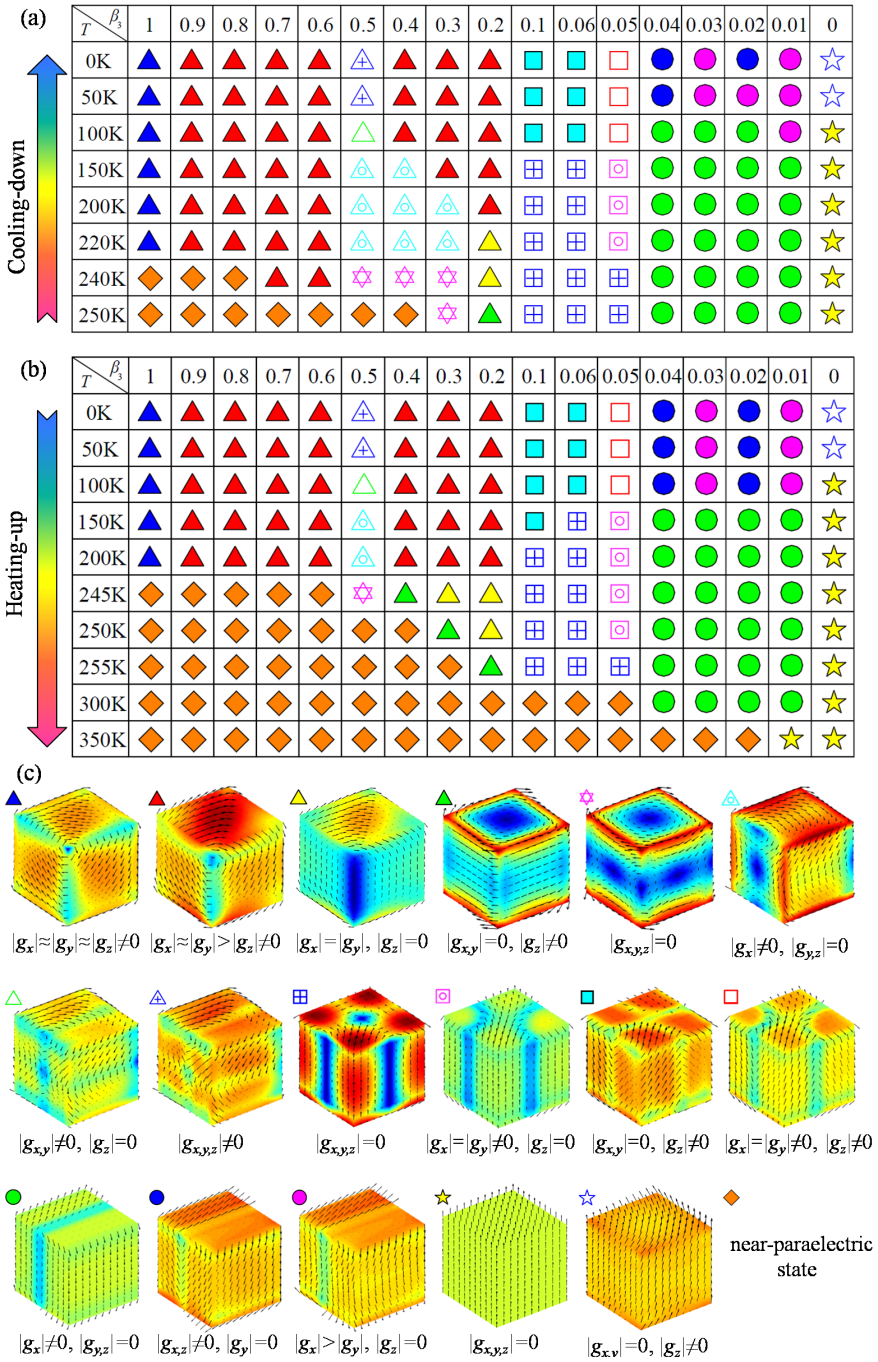
Phase diagram of the equilibrium domain patterns in a nanodot. In (a) cooling-down and (b) heating-up processes under different surface charge screening boundary conditions. (c) The obtained equilibrium domain patterns and their denoted symbols.

**Figure 4:**
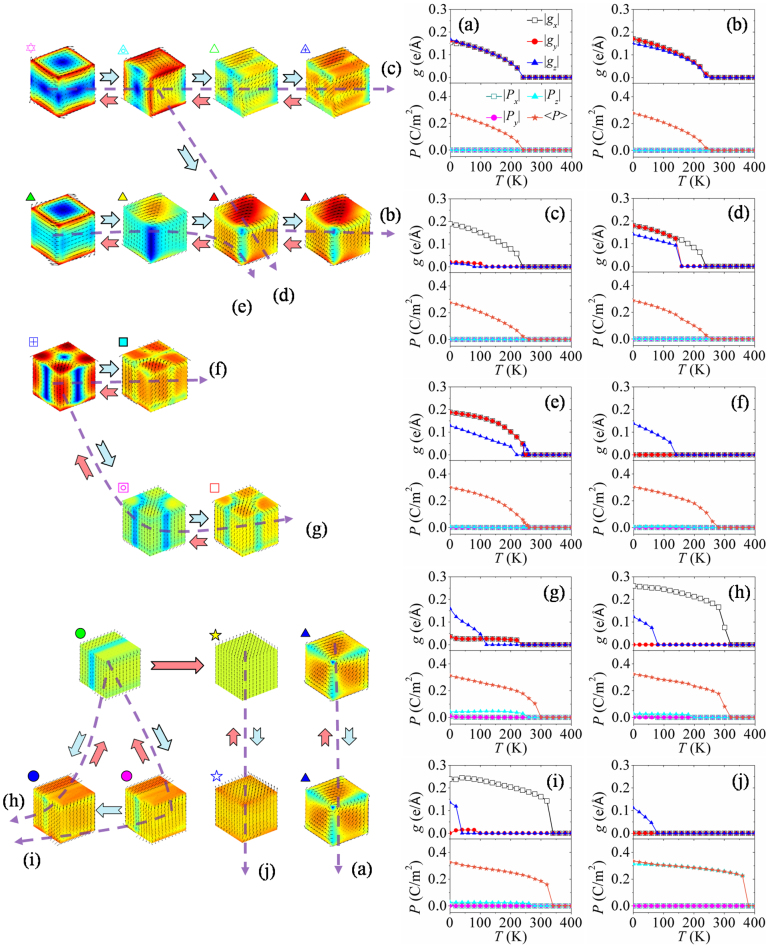
Evolution of toroidal moments and polarizations (right panels) of a nanodot. In cooling-down process under different charge screening conditions with screening factor (a) *β*_3_ = 1, (b) *β*_3_ = 0.7, (c) *β*_3_ = 0.5, (d) *β*_3_ = 0.4, (e) *β*_3_ = 0.2, (f) *β*_3_ = 0.1, (g) *β*_3_ = 0.05, (h) *β*_3_ = 0.04, (i) *β*_3_ = 0.02, and (j) *β*_3_ = 0. Left panels depict the evolution paths of equilibrium domain pattern during cooling-down process (azury arrow) and heating-up process (pink arrow) under different charge screening conditions corresponding to the right panels.

In the following, we would like to make a comprehensive research on the evolution of domain pattern in nanodots during cooling-down process under various surface charge screening conditions. Similar with the simulation shown in Figures 2a and 2b, for each cooling-down process, a random perturbation of polarization field is introduced to initiate the polarization evolution at high temperature. Once a stable domain pattern is formed below paraelectric-ferroelectric transition temperature, it is used as the initial domain pattern for the next temperature step. The temperature step is chosen to be 5 K in the cooling-down process. We analyze the evolution of domain pattern at each temperature and under each surface charge screening condition. To be illustrative, in Figure 3a we plot a phase diagram of cooling-down process, which depicts the equilibrium domain pattern of the nanodot as a function of temperature and charge screening factor, with each type of domain pattern represented by a specific symbol as shown in Figure 3c. The typical transformation paths of domain pattern during the cooling-down process are plotted in Figure 4 (indicated by the azury arrows), together with the corresponding evolution graphs of toroidal moment and polarization.

From the cooling-down phase diagram, we can see that fruitful domain patterns and transformation paths can be obtained by adjusting surface charge screening condition on the nanodot. This phase diagram can be roughly characterized by three main regions. The first region is the one between *β*_3_ = 0.2 and *β*_3_ = 1, where the equilibrium domain patterns are all vortex domain patterns. To be specific, in the process of cooling-down under ideal open-circuit condition (*β*_3_ = 1) and near open-circuit (*β*_3_ = 0.6 ~ 0.9) boundary condition, the nanodot adopts rhombohedral and orthorhombic-like vortex domain patterns respectively, with the corresponding toroidal moment components and the average magnitudes of polarization gradually increasing. The typical transformation paths are shown in Figure 4a (*β*_3_ = 1) and 4b (*β*_3_ = 0.7). Interestingly, when the charge screening factor *β*_3_ is between 0.3 and 0.5, the nanodot adopts a vortex domain pattern with opposite rotation directions at the top and bottom surfaces at *T* ≥ 240 K. At the conjunction of the two vortices, there are also four small vortices locating at middle of the edges of nanodot. As the nanodot is further cooled down, the domain pattern would change into three similar vortices in succession with |*g_x_*| ≠ 0 and 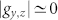 until 0 K at *β*_3_ = 0.5 (see also in Figure 4c). The evolution process from the 2-vortices domain pattern to that with |*g_x_*| ≠ 0 and |*g_y,z_*| = 0 can be seen in Supplementary Figure S1(a) on line. Different from the evolution path at *β*_3_ = 0.5, the high temperature domain pattern would gradually evolve into orthorhombic-like vortex domain pattern as temperature decreases when the nanodot is under charge screening *β*_3_ = 0.3 (*T* < 195 K) and *β*_3_ = 0.4 (*T* < 150 K). The detail of this evolution process is shown in Supplementary Figure S1(b) on line. This orthorhombic-like vortex domain pattern possesses smaller toroidal moment component |*g_z_*| than the other two components, as can be seen in Figure 4d for *β*_3_ = 0.4. During the cooling-down process at charge screening *β*_3_ = 0.2, the nanodot would first form a vortex domain pattern (|*g_x,y_*| = 0 and |*g_z_*| ≠ 0) with the dipoles lying in *x*-*y* plane as shown in Figure 4e. Then the domain pattern transforms into a perfect orthorhombic vortex domain pattern (|*g_x_*| = |*g_y_*|,|*g_z_*| = 0) at 240 K (see Supplementary Figure S1(c) on line) and orthorhombic-like vortex domain pattern afterwards, similar with the above mentioned ones.

In the second region of the phase diagram in Figure 3, i.e., when the surface charge screening factor is between *β*_3_ = 0.05 and *β*_3_ = 0.1, the nanodot would form a highly symmetric 4-vortices domain pattern (|*g_x,y,z_*| = 0) at high temperature. The toroidal axes of the vortices are along 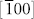, [100], 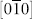 and [010] directions. When the temperature further decreases, the domain pattern transforms into tilted 4-vortices domain patterns with |*g_z_*| ≠ 0 near 0 K, as depicted in Figures 4f (*β*_3_ = 0.1) and 4g (*β*_3_ = 0.05). In this region, the toroidal moment components |*g_x_*| and |*g_y_*| of the domain patterns are always identical. They are null when *β*_3_ is between 0.06 and 0.1 and are nonzero when *β*_3_ = 0.05 below 230 K.

The third region of phase diagram is *β*_3_ < 0.05, and its characteristic is no obvious dipole vortex in the domain patterns. Particularly, the nanodot would first adopt 180°-like domain pattern (with most dipoles almost aligning along *z*-direction and |*g_x_*| ≠ 0, |*g_y,z_*| = 0) during the cooling-down process (0.01 ≤ *β*_3_ ≤ 0.04), because the significant surface charge screening can largely reduce the depolarization field along *z*-direction. Then the domain pattern would transform into a tilted 180°-like domain pattern with only |*g_y_*| = 0 or |*g_z_*| = 0 at 0 K. This transformation path of domain pattern can be clearly seen in Figure 4h, where the 180°-like domain pattern with |*g_x_*| ≠ 0 would evolve into the tilted 180°-like domain pattern with |*g_y_*| being null directly at *β*_3_ = 0.04. However, when the screening factor *β*_3_ is 0.02, the domain pattern would experience an intermediate state with a small |*g_y_*| shown in Figure 4i. Under the complete charge screening boundary condition, a polar single-domain pattern (|*g_x_*| = |*g_y_*| = |*g_z_*| = 0 and 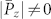) appears at 375 K due to the strongest charge screening effect, and the nanodot keeps this domain pattern until *T* = 80 K (Figure 4j), with the average magnitude of polarization increasing. If the temperature continues decreasing below 80 K, a single-domain-like pattern (|*g_x,y_*| = 0, |*g_z_*| ≠ 0) with average polarization component 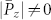 is formed because of the relatively large polarization at low temperature. From the polarization evolution curves of the domain patterns as shown in Figure 4, we can also find that the average magnitude of polarization <*P*> increases gradually from zero when the temperature decreases through the paraelectric-ferroelectric transition point with screening factor *β*_3_ being large (see Figures 4a–g). Meanwhile, <*P*> increases abruptly from zero when *β*_3_ is near zero, especially in Figures 4h–j. This means that nearly short-circuit condition, the paraelectric-ferroelectric phase transition of the nanodot exhibits a first-order feature, and this can be gradually adjusted into second-order feature by decreasing the charge screening to open-circuit condition.

### Nanodot in heating-up process under various charge screening conditions

In this part, we focus on the domain structure evolution of nanodots in heating-up process under various charge screening conditions, which is in opposite direction of cooling-down process (see Figure 3a). The differences of domain patterns and transformation paths between heating-up and cooling-down processes under the same surface charge screening condition are compared in the following. The initial domain patterns are those at 0 K obtained from cooling-down process under corresponding surface charge screening conditions, which are already discussed in the previous section (see Figure 3a). The temperature step is chosen to be 5 K similar to the cooling-down process.

From the temperature and charge screening phase diagram of heating-up process in Figure 3b, we find that this process is much similar to that of cooling-down process. Nevertheless, some kinds of domain patterns would maintain stable in a larger range of temperature during the heating-up process, indicating a hysteresis of transformation. For example, the orthorhombic-like vortex domain pattern of nanodot under charge screening *β*_3_ = 0.4 would maintain stable from 0 K to 200 K during heating-up process, whereas this domain pattern keeps stable only in range of 0 ~ 100 K during the process of cooling-down at the same *β*_3_. It is also interesting to see that in some range of charge screening, e.g., *β*_3_ = 0.3 or 0.4, the domain patterns at low temperature do not evolve in exactly the opposite directions that appear in the cooling-down process. As a consequence, we can see that some of the domain patterns obtained in the heating-up process are absent in the cooling-down process. For example, the perfect orthorhombic vortex domain pattern (|*g_x_*| = |*g_y_*| ≠ 0, |*g_z_*| = 0) that appears under condition *β*_3_ = 0.3 during heating-up process does not appear in the cooling-down process as shown in Figures 3a and 3b. This difference can be also seen from the evolution paths indicated by azury and pink arrows shown in Figure 4. The vortex domain pattern with toroidal moment component |*g_x_*| ≠ 0, |*g_y,z_*| = 0 can transform into the orthorhombic-like vortex domain pattern in the cooling-down process as depicted in Figure 4d (follow the azury arrows), but during the opposite process of heating-up the nanodot does not exhibit such a transformation of domain patterns. When the screening factor *β*_3_ is 0.02, the toroidal moment component |*g_y_*| of the domain patterns always keeps null with the temperature increasing. However, a 180°-like domain pattern with a small |*g_y_*| is formed in the cooling-down process with *β*_3_ being 0.02 (5 K < *T* < 100 K).

**Figure 5:**
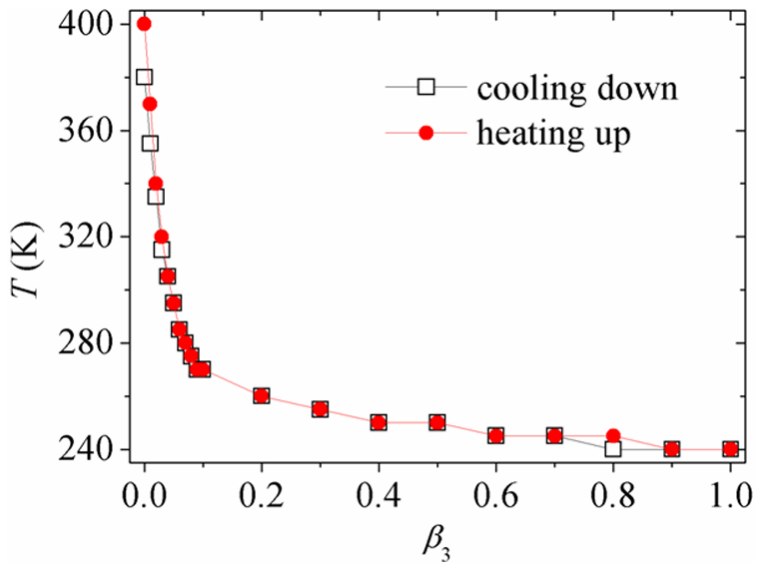
Paraelectric-ferroelectric phase transition temperature. Paraelectric-ferroelectric phase transition temperature of a nanodot in processes of cooling-down and heating-up as a function of charge screening.

To see the impact of surface charge screening on the cooling-down and heating-up phase transition behaviors more clearly, paraelectric-ferroelectric phase transition temperatures as functions of charge screening of the two processes are drawn in Figure 5. It can be seen that the phase transition temperatures of cooling-down process and heating-up process are almost the same under most of the charge screening conditions, indicating a second-order feature of the phase transition. However, under near short-circuit charge screening conditions, especially when *β*_3_ < 0.04, the difference of heating-up and cooling-down phase transition temperatures becomes significant, depicting an increasing first-order feature, and the maximum difference of phase transition temperature between these two processes would reach 20 K at *β*_3_ = 0. Compared with the heating-up and cooling-down phase diagrams of the domain patterns (see Figure 3), it can be seen that the transformation of domain patterns also exhibits an increasing first-order feature as the charge screening increases.

### Nanodot in increasing charge screening process at different temperatures

In the previous calculations, we have obtained fruitful domain patterns controlled by surface charge screening during temperature changing processes. Considering the possible applications where the ferroelectric nanodot is under variable charge screening condition, in the following we would like to explore the effect of variable charge screening on the formed domain pattern of the nanodot at fixed temperatures. First of all, the domain patterns at different temperatures obtained from the process of cooling-down under open-circuit boundary condition are used as the initial patterns of the nanodot for the polarization evolution under the next charge screening condition. Then the equilibrium domain patterns are used as the initial domain patterns of the next charge screening condition. The charge screening *β*_3_ increases by a step as small as 0.01.

**Figure 6:**
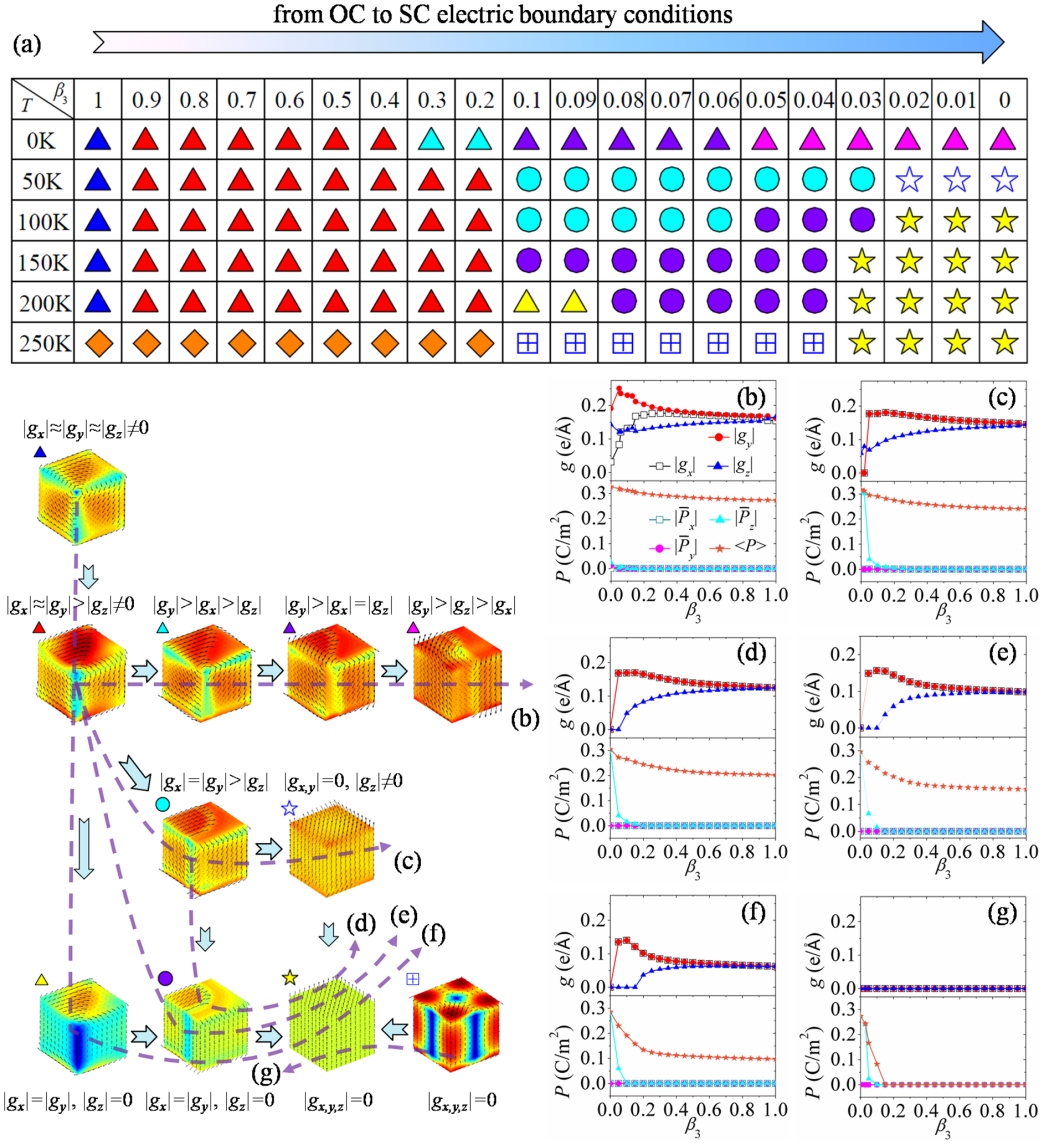
Domain pattern evolution of a nanodot in increasing charge screening process. (a) Phase diagram of the equilibrium domain patterns as a function of charge screening and temperature. Toroidal moments, polarizations and equilibrium domain patterns as functions of charge screening at the temperature of (b) 0 K, (c) 50 K, (d) 100 K, (e) 150 K, (f) 200 K, and (g) 250 K.

During the charge screening increasing process, the evolution of domain pattern of the nanodot at fixed temperatures is clearly seen in the phase diagram shown in Figure 6a. Typical transformation paths of domain pattern are also plotted in Figures 6b–g, together with the corresponding evolution graphs of toroidal moment and polarization. Compared with the phase diagrams obtained in cooling-down/heating-up processes (see Figures 3a and 3b), it can be seen that the phase diagram of increasing charge screening process is much different, particularly in the near short-circuit boundary condition region. When under ideal or near open-circuit boundary condition, the nanodot adopts rhombohedral or orthorhombic-like vortex domain patterns. When the charge screening factor *β*_3_ is less than ~0.3 at 0 K, the vortex axis of the domain pattern tilts away from the bulk diagonal lines, with the toroidal moment component |*g_x,y_*| firstly increasing and then decreasing, and with |*g_z_*| firstly decreasing and then increasing, as indicated in Figure 6b. In this process, the final domain pattern at *β*_3_ ~ 0 has its vortex axis tilting to the *y*-*z* plane and |*g_y_*| > |*g_z_*| > |*g_x_*|. Moreover, the average polarization keeps near-null at 0 K.

In case of *T* = 50 K as shown in Figures 6a and 6c, when *β*_3_ deceases to ~0.2, the orthorhombic-like vortex domain pattern would transform into a novel domain pattern which does not appear during the cooling-down/heating-up processes. For this domain pattern, the dipole vortices tilt to the *z*-axis and their cores no longer align along a straight line (e.g., along the bulk diagonal) but a curved line. More importantly, the domain pattern exhibits a net polarization, i.e., 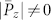. For this reason, in the following we would like to call this domain pattern as polar vortex domain pattern. The nanodot would form a single-domain-like pattern with small toroidal moment |*g_z_*| at charge screening *β*_3_ ≤ 0.02. In this transformation process, toroidal moment components |*g_x_*| and |*g_y_*| are equal throughout the process of charge screening increasing. When *β*_3_ is less than ~0.2, the average polarization 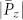 would increase gradually and reach to 0.31 C/m^2^ under the ideal short-circuit boundary condition.

For the nanodot at *T* = 100 K, the transformation behavior of domain pattern is similar with that at *T* = 50 K until charge screening *β*_3_ ≤ 0.05, in which toroidal moment component |*g_z_*| becomes zero (Figure 6d). Actually for this condition, the dipole vortices of the polar vortex domain pattern are exactly parallel to *z*-axis when the charge screening factor *β*_3_ is in-between 0.05 and 0.03. After that, a perfect single-domain pattern is formed as the charge screening increases. Compared with the transformation at 100 K, when the temperature reaches 150 K, the domain structure would transform from the orthorhombic-like vortex domain pattern to the polar vortex domain pattern with toroidal moment component |*g_z_*| equal to zero directly for charge screening *β*_3_ = 0.14 shown in Figure 6e. Interestingly, a perfect orthorhombic vortex domain pattern appears as an intermediate domain pattern at 200 K with screening factor *β*_3_ between 0.16 and 0.09 (Figure 6f).

If the temperature further increases to 250 K, which is near the paraelectric-ferroelectric transition temperature, the nanodot would be in paraelectric state when screening factor *β*_3_ is more than 0.11(see Figure 6a). Nevertheless, as the charge screening increases, the 4-vortices domain pattern emerges under near short-circuit boundary condition (0.04 ≤ *β*_3_ ≤ 0.11), with the three toroidal moment components being null as shown in Figure 6g. Then the domain pattern of the nanodot transforms into a perfect single-domain pattern at *β*_3_ = 0.03 (the detailed evolution is depicted in Supplementary Figure S1(d) on line). Moreover, we can see from Figure 6 that the nanodot would form perfect single-domain pattern under the near short-circuit boundary condition in increasing charge screening process if the temperature is more than 100 K, indicating the transformation of vortex domain pattern to polar domain pattern.

### Nanodot in decreasing charge screening process at different temperatures

**Figure 7:**
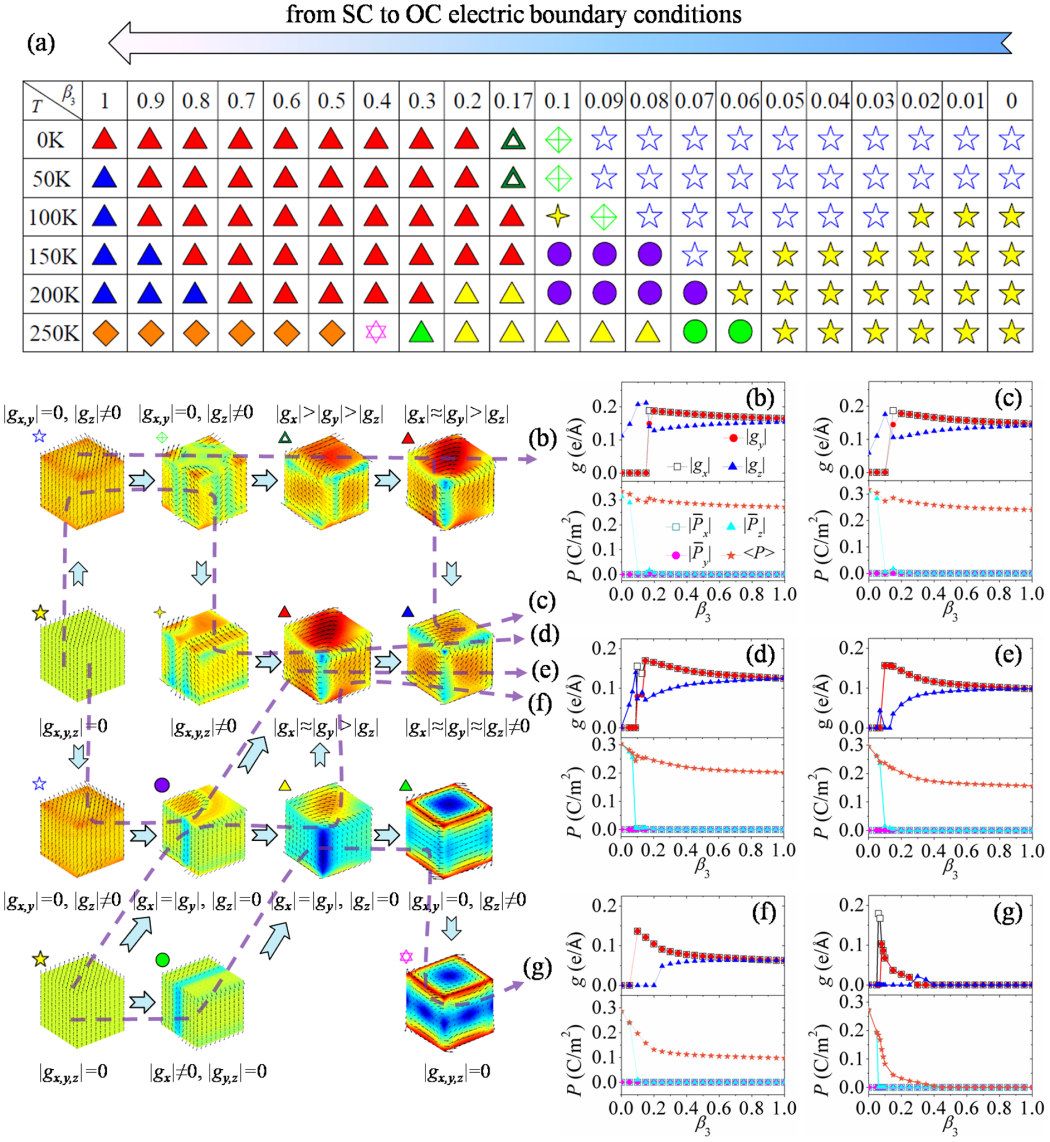
Domain pattern evolution of a nanodot in decreasing charge screening process. (a) Phase diagram of the equilibrium domain patterns as a function of charge screening and temperature. Toroidal moments, polarizations and equilibrium domain patterns as functions of charge screening at the temperature of (b) 0 K, (c) 50 K, (d) 100 K, (e) 150 K, (f) 200 K, and (g) 250 K.

To understand the charge screening effect more clearly, we analyze the transformation of domain patterns in decreasing charge screening process at fixed temperatures as shown in Figure 7a to compare with the reverse process of increasing charge screening in the previous simulations. The corresponding transformation paths of domain pattern and their evolution graphs of toroidal moment and polarization are plotted in Figures 7b–g. Similar with the increasing charge screening process, we first obtain domain patterns of the nanodot at different temperatures from short-circuit boundary condition during temperature cooling-down process, and then use these domain patterns as initial domain patterns for the polarization evolution under next charge screening condition. The charge screening *β*_3_ decreases by a step as small as 0.01.

When the temperature is 0 K, the initial single-domain-like pattern of the nanodot keeps stable until *β*_3_ = 0.09, which is much different from the reverse process. As the charge screening decreases, the domain pattern would involve into a novel domain pattern with 90° domain walls for *β*_3_ in-between 0.1 and 0.16. For this domain pattern, we find that the domains are rotated around the *z*-axis, leading to |*g_x,y_*| = 0, |*g_z_*| ≠ 0. The detailed evolution process can be found in Supplementary Figure S1(e) on line. Then a vortex domain pattern with |*g_x_*| > |*g_y_*| > |*g_z_*| (0.17 ≤ *β*_3_ ≤ 0.19) is formed and shown in Figure 7b. For the charge screening *β*_3_ from 0.2 to 1, the nanodot always keeps orthorhombic-like vortex domain pattern. The transformation of the domain patterns at 50 K is much similar to that at 0 K. However, the nanodot would form rhombohedral vortex domain pattern under open-circuit boundary condition, which is indicated in Figure 7c. When the temperature is 100 K, the initial domain structure is polar single-domain pattern, which would transform into single-domain-like pattern and then into the domain pattern with 90° domain walls as the charge screening decreases. For *β*_3_ in-between 0.1 and 0.13, another domain pattern with 90° domain walls and |*g_x,y,z_*| ≠ 0 appears (see Figure 7d), which is less symmetric than the previous one. The details of this evolution process are depicted in Supplementary Figure S1(f) on line. Orthorhombic-like and rhombohedral vortex domain patterns appear again in succession as the boundary condition is further away from short-circuit condition.

At *T* = 150 K, the nanodot forms a polar vortex domain pattern (|*g_x_*| = |*g_y_*|, |*g_z_*| = 0, 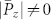) as *β*_3_ increases from 0.08 to 0.13, with the cores of dipole vortices along a curved line and the dipole vortices parallel to the *z*-axis. From Figure 7e, we can see that the toroidal moment components |*g_x_*| and |*g_y_*| of this vortex domain pattern are equal with each other, and its average polarization component 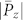 is not zero. For the nanodot at 200 K, this polar vortex domain pattern evolves from the perfect polar single-domain pattern and directly transforms into orthorhombic vortex domain pattern, which appears within *β*_3_ = 0.13 to *β*_3_ = 0.2 (Figure 7f). As the charge screening continues to decrease, the orthorhombic vortex domain pattern first transforms into orthorhombic-like vortex domain pattern and then into rhombohedral vortex domain pattern at 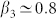. When the temperature reaches 250 K, the transformation of domain patterns is much different from those at the above temperatures. At the beginning, the single-domain pattern transforms into 180°-like domain pattern as the screening factor *β*_3_ increases to 0.06, and it becomes orthorhombic vortex domain pattern at *β*_3_ = 0.08. As *β*_3_ in-between 0.26 and 0.38, the nanodot adopts a vortex domain pattern along <100> direction with |*g_x_*| = |*g_y_*| = 0 shown in Figure 7g. Then the nanodot adopts a vortex domain pattern with opposite rotation directions at the top and bottom surfaces as the charge screening decreases. The evolution of this transformation can be found in Supplementary Figure S1(g) on line. Finally, the nanodot turns into paraelectric state from *β*_3_ = 0.41. Basing on these results, we can find that the 4-vortices domain pattern does not appear in this process, which is quite special and appears in increasing charge screening process at *T* = 250 K (see Figures 6a and 6g).

## Discussion

In summary, we have systematically investigated the transformations of vortex domain structure in a ferroelectric nanodot under the control of surface charge screening and temperature. Through adjusting the electric boundary condition or temperature, we have shown that the nanodot can form fruitful vortex domain structures. Four significant charge screening and temperature phase diagrams are summarized, together with the typical evolution paths of domain patterns. It is found that the transformations of domain structures in the processes of heating-up and cooling-down under the same charge screening boundary condition are similar approximately. However, the phase diagrams under increasing and decreasing charge screening at the fixed temperatures are significantly different from each other, indicating that the initial domain structure plays an important role in these transformations. In addition, when the nanodot is under the near short-circuit boundary condition or at high temperature near paraelectric-ferroelectric phase transition temperature, more various domain patterns can appear due to the strong charge screening effect and small polarization. These results reveal the prospective application of charge screening and temperature in the design of nanoscale multifunctional devices.

## Methods

Our phase-field simulation of ferroelectric domain structure is based on a Landau-Devonshire-Ginzburg free-energy, which takes into account the effects of inhomogeneous electromechanical fields. Finite element methods are adopted to solve the electrostatic and mechanical equilibrium equations. The evolution of polarization field is solved by discretizing the time-dependent Ginzburg-Landau equation to determine kinetics of polarization field. In the calculations, we assume that the system reaches the electrostatic and mechanical equilibrium instantaneously once the spontaneous polarization distribution is changed. To get the effect of charge screening and temperature on the vortex domain structure of the ferroelectric nanodot, a meshing of 10 × 10 × 10 elements with interval scale being equal to 0.4 nm is used to simulate a cubic nanodot, and the charge screening is at the top and bottom surfaces. A detailed description of the phase-field approach is provided in the Supplementary Information on line.

## Author Contributions

Y.Z. initiated and performed this work and manuscript. B.W. suggested the principle idea. W.J.C. and Y.Z. conceived and designed the basic idea, structures and simulations. C.M.W. and W.J.C. performed the simulations. Y.Z., C.M.W., W.J.C., B.W., D.C.M., C.H.W. and J.Y.L. analyzed the results of simulations. Y.Z., B.W., C.M.W. and W.J.C. co-wrote the manuscript. All authors contributed to discussion and reviewed the manuscript.

## Supplementary Material

Supplementary Information Supplementary Information
