# Supplementary material for: Controllability of Vortex Domain Structure in Ferroelectric Nanodot: Fruitful Domain Patterns and Transformation Paths
Source: Sci Rep. 2014 Feb 4;4:3946. doi: 10.1038/srep03946 (PMC3912473; doi:10.1038/srep03946)
Supplement: Supplementary Information [file srep03946-s1.pdf]

Controllability of Vortex Domain Structure in Ferroelectric Nanodot by Surface Charge Screening: Fruitful Domain Patterns and Transformation Paths

C. M. Wu,[1] W. J. Chen,[1] Yue Zheng[1,*], D. C. Ma,[2] B. Wang[1,*], J. Y. Liu[1] and C. H. Woo[3]

[1]State Key Laboratory of Optoelectronic Materials and Technologies, Micro&Nano Physics and Mechanics Research Laboratory, School of Physics and Engineering, Sun Yat-sen University, Guangzhou 510275, China, [2]Sino-French Institute of Nuclear Engineering and Technology, Zhuhai Campus, Sun Yat-sen University, Zhuhai 519082, China, and [3] Department of Physics and Materials Science, City University of Hong Kong, Hong Kong SAR, China

Supplementary Information

In this research, we take the free-standing $BaTiO_3$ nanodot as example to demonstrate the effect of charge screening and temperature on the vortex domain structure of the ferroelectric nanostructures. The temperature regions and polarization values of each phase for bulk $BaTiO_3$ are listed in Table S1 to compare with those in $BaTiO_3$ nanodot.

Table S1 | Temperature regions and polarization values of each phase for bulk $BaTiO_3$.

Phase	Temperature Region (K)	Polarization (C/m^2)			
rhombohedral	0~202	P_1=0.237	P_2=0.237	P_3=0.237	(T=100K)
orthorhombic	202~281	P_1=0.229	P_2=0	P_3=0.229	(T=250K)
tetragonal	281~398	P_1=0	P_2=0	P_3=0.259	(T=300K)
cubic	≥398	P_1=0	P_2=0	P_3=0	(T=500K)

The free energy model we use is from ref.5.

Phase-Field Simulations

The ferroelectric domain structures in the nanodot are predicted by the phase-field simulation, which is executed by the time-dependent Ginzburg-Landau (TDGL) equation, i.e.,

$$\frac{\partial P_i}{\partial t} = -M \frac{\delta F}{\delta P_i} \qquad (i = 1, 2, 3) \tag{S1}$$

where F is the total free energy of the system, t is time, M is the kinetic coefficient related to the domain wall mobility, and P_i are spontaneous polarization components. In this approach, we use the spontaneous polarization $\mathbf{P} = (P_1, P_2, P_3)$ as order parameter. Therefore, the electric displacement field **D** is expressed as $\mathbf{D}=\boldsymbol{\varepsilon}_\mathbf{b}\mathbf{E}+\mathbf{P}$, where **E** is the electric field and $\boldsymbol{\varepsilon}_\mathbf{b}$ is the background dielectric constant tensor.[1-3] The background material is the paraelectric phase of a cubic structure, so the background dielectric constants along the three axial directions are the same, i.e.,

$\varepsilon_b = \varepsilon_{11b} = \varepsilon_{22b} = \varepsilon_{33b}$.

In our calculations, the inhomogeneous electromechanical fields and surface are considered to investigate the domain structures in a ferroelectric nanodot. Accordingly, the total free energy of the ferroelectric nanodot can be expressed as a sum of the Landau-Devonshire energy, gradient energy, electrostatic energy, and surface energy, i.e.,

$$F = \int_V (f_{\mathrm{LD}} + f_{\mathrm{grad}} + f_{\mathrm{elec}}) dV + \int_S f_{\mathrm{surf}} dS \tag{S2}$$

where V and S are the volume and the surface of the nanodot, f_{LD}, f_{grad}, f_{elec} and f_{surf} are densities of the Landau-Devonshire energy, gradient energy, electric energy, and surface energy, respectively.

An eighth-order polynomial of the modified Landau-Devonshire energy density is written as[4-6]

$$\begin{aligned} f_{\mathrm{LD}} &= \alpha_1(P_1^2+P_2^2+P_3^2)+\alpha_{11}(P_1^4+P_2^4+P_3^4)+\alpha_{12}(P_1^2P_2^2+P_2^2P_3^2+P_1^2P_3^2) \\ &+\alpha_{111}(P_1^6+P_2^6+P_3^6)+\alpha_{112}[P_1^2(P_2^4+P_3^4)+P_2^2(P_1^4+P_3^4)+P_3^2(P_1^4+P_2^4)] \\ &+\alpha_{123}P_1^2P_2^2P_3^2+\alpha_{1111}(P_1^8+P_2^8+P_3^8)+\alpha_{1112}[P_1^6(P_2^2+P_3^2)+P_2^6(P_1^2+P_3^2) \\ &+P_3^6(P_1^2+P_2^2)]+\alpha_{1122}(P_1^4P_2^4+P_2^4P_3^4+P_1^4P_3^4)+\alpha_{1123}(P_1^4P_2^2P_3^2+P_2^4P_1^2P_3^2 \\ &+P_3^4P_1^2P_2^2)-Q_{11}(\sigma_{11}P_1^2+\sigma_{22}P_2^2+\sigma_{33}P_3^2)-Q_{12}[\sigma_{11}(P_2^2+P_3^2)+\sigma_{22}(P_1^2+P_3^2) \\ &+\sigma_{33}(P_1^2+P_2^2)]-Q_{44}(\sigma_{12}P_1P_2+\sigma_{13}P_1P_3+\sigma_{23}P_2P_3)-\tfrac{1}{2}s_{11}(\sigma_{11}^2+\sigma_{22}^2+\sigma_{33}^2) \\ &-s_{12}(\sigma_{11}\sigma_{22}+\sigma_{22}\sigma_{33}+\sigma_{11}\sigma_{33})-\tfrac{1}{2}s_{44}(\sigma_{12}^2+\sigma_{23}^2+\sigma_{13}^2) \end{aligned} \tag{S3}$$

where $\alpha_1 = (T - T_0)/(2\varepsilon_0 C_0)$ is the dielectric stiffness, T_0 and C_0 are the Curie-Weiss temperature and Curie-Weiss constant, respectively, ε_0 is the vacuum permittivity, and T is the temperature. α_{ij}, α_{ijk} and α_{ijkl} are the higher-order dielectric stiffness coefficients. Q_{ij} and s_{ij} are the electrostrictive and elastic compliance coefficients, respectively. The stress components σ_{ij} include applied and internal stresses induced by spontaneous strains, and should be determined by the

mechanical equilibrium equation, i.e., $\sigma_{ij,j}=0$, together with the mechanical boundary condition $\sigma_{ij}n_j=0$ at the surface, wherein the comma in the subscript denotes the spatial differentiation and n_j is the *j*-component of the unit vector normal to the surface.

The gradient energy represents the spatial variation of the polarization in ferroelectric nanodot. To the lowest order Taylor expansion for simplicity, the gradient energy density f_{grad} is

$$\begin{aligned} f_{\text{grad}}(P_{i,j}) = {} & \tfrac{1}{2}G_{11}(P_{1,1}^2+P_{2,2}^2+P_{3,3}^2)+G_{12}(P_{1,1}P_{2,2}+P_{2,2}P_{3,3}+P_{1,1}P_{3,3}) \\ & +\tfrac{1}{2}G_{44}[(P_{1,2}+P_{2,1})^2+(P_{2,3}+P_{3,2})^2+(P_{1,3}+P_{3,1})^2] \\ & +\tfrac{1}{2}G_{44}^{'}[(P_{1,2}-P_{2,1})^2+(P_{2,3}-P_{3,2})^2+(P_{1,3}-P_{3,1})^2] \end{aligned} \quad \text{(S4)}$$

in which G_{11}, G_{12}, G_{44} and $G_{44}^{'}$ are gradient energy coefficients.

When there is not any external electric field, the electric field of the nanodot is the depolarization field, which is induced by spatially inhomogeneous spontaneous polarization and incomplete screening of the polarization charges at the surface and interface of the nanodot. The electric energy density is calculated by[1, 2, 7]

$$f_{\text{elec}} = -P_1E_1 - P_2E_2 - P_3E_3 - \tfrac{1}{2}\varepsilon_b\left(E_1E_1+E_2E_2+E_3E_3\right) \quad \text{(S5)}$$

The electric field **E** can be obtained by solving the electrostatic equilibrium equation, i.e., $\mathbf{D}_{i,i}=(\varepsilon_{\text{b}}\mathbf{E}+\mathbf{P})_{i,i}=0$ with the boundary condition $D_in_i=0$ for a given polarization field in a body-charge-free system.

An additional surface energy is used to describe the effect of inhomogeneous polarization from the truncation near surfaces. Accordingly, the surface energy density is given by

$$f_{\text{surf}} = \frac{D_{11}P_1^2}{2\delta_1}+\frac{D_{22}P_2^2}{2\delta_2}+\frac{D_{44}P_3^2}{2\delta_3} \quad \text{(S6)}$$

in which δ_i are the extrapolation length,[8] and D_{ij} are the material coefficients related to the gradient energy coefficients and surface orientation.

Values of the expansion coefficients of the eighth-order Landau-Devonshire potential, electrostrictive coefficients, elastic properties,[5, 9, 10] and extrapolation length[11] in our simulation are listed in Ref. 12.

Analysis of Domain Morphology Evolution

To understand the changing process of domain pattern in the paper more clearly, seven typical evolutions of domain morphologies in ferroelectric nanodot are shown in Figure S1. The "step" here manifests the time step in the simulation calculations. From Figure S1(a), we can see that the center of the initial 2-vortices domain pattern at the top surface moves to the edge of the nanodot gradually. Meanwhile, the middle region of the domain pattern at the side surface moves to the small vortex which locates at the junction of the two vortices, and then they coalesce together to lose the small vortex. Finally, the domain pattern with toroidal moment component $|g_x| \neq 0$ would be stable after about 2.3e5 time steps. The evolution from the vortex domain pattern which is formed at last in Figure S1(a) to orthorhombic-like vortex domain pattern is indicated in Figure S1(b). The domain walls at the side surface of the domain pattern in the nanodot move towards the edge along *z*-direction. After orthorhombic vortex domain pattern at about 5.7e5 time steps, the axis of the domain pattern turns from <110> direction towards <111> direction gradually and achieves the final stable state at near 6e5 time steps. The evolution in Figure S1(c) is about the

transformation from a vortex domain pattern with <100> direction to one with <110> direction. At the beginning, the center of the vortex domain at the outside surface of the nanodot is at the middle of the top/bottom surface. Then it moves to the edge of that surface and reaches the corner of the nanodot at about 1.5e5 time steps with axis along near <111> direction. After that, the axis of the vortex domain pattern moves towards *x*-*y* plane, and finally the domain pattern transforms into an orthorhombic vortex domain pattern. For the 4-vortices domain pattern, the evolution to single-domain structure is completed by moving domain walls to two parallel edges of the nanodot and disappearing at last, as seen in Figure S1(d). For the evolution in Figure S1(e), the initial single-domain-like structure would rotate the direction of the polarization at the edges of the nanodot gradually and achieve a stable domain structure with near 90° domain walls. As we can see in Figure S1(f), the final domain pattern is formed by rotating the direction of the polarization at the middle of the *x*-*z* plane in the initial domain pattern of the nanodot. In Figure S1(g), the initial vortex domain pattern with axis along <100> direction would decrease the polarization gradually, especially the half bottom of the nanodot to near zero. Then four small vortices are formed at the corners of nanodot and move towards the middle of the edges. Meanwhile, the polarization in the nanodot would increase. The 4-vortices domain pattern is formed after about 10^6 time steps.

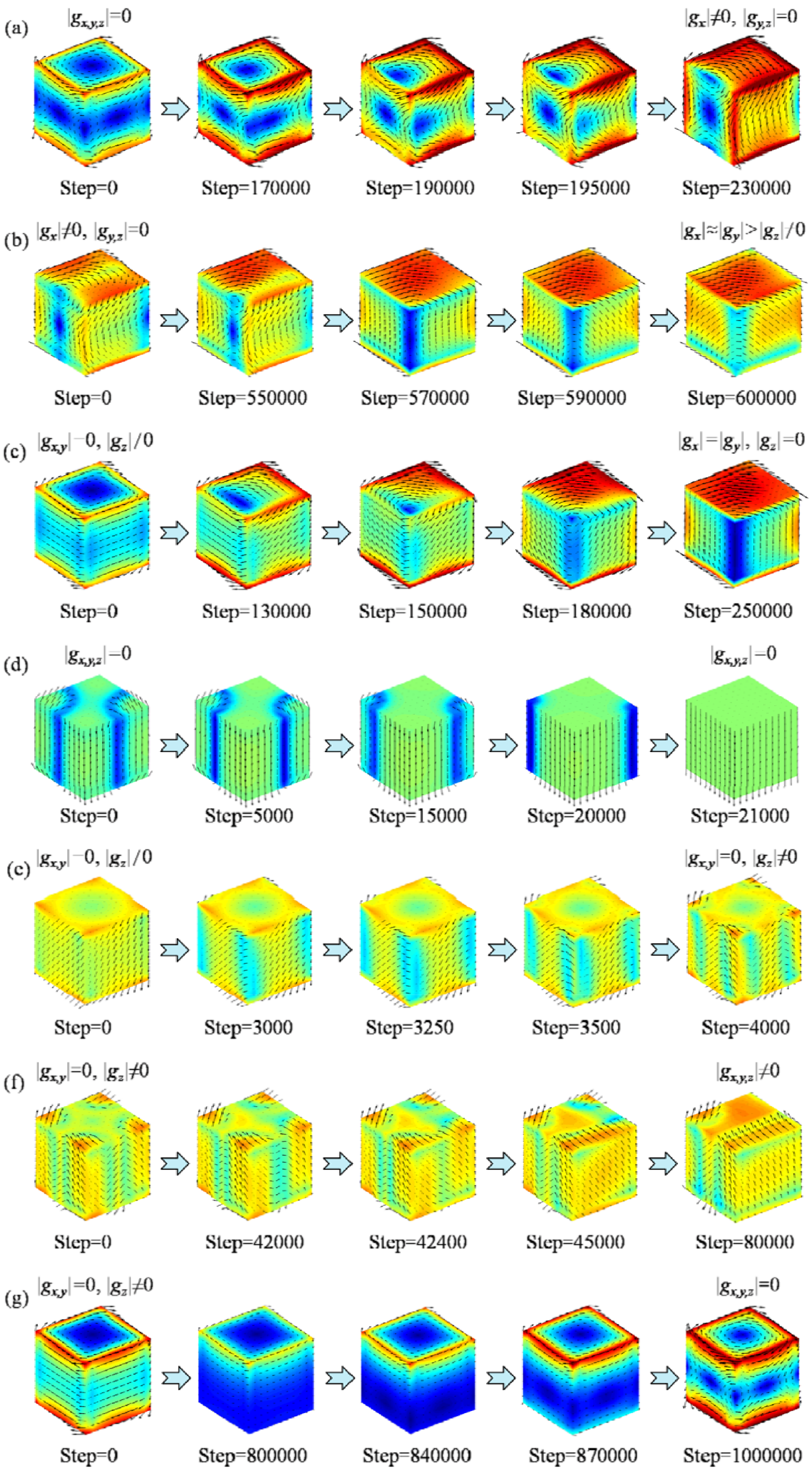

Figure S1. Evolution of domain morphology in nanodot.

1. Zheng, Y.; Woo, C. H., Thermodynamic modeling of critical properties of ferroelectric superlattices in nano-scale. *Applied Physics A* 2009, 97, 617-626.
2. Woo, C. H.; Zheng, Y., Depolarization in modeling nano-scale ferroelectrics using the Landau free energy functional. *Applied Physics A* 2008, 91, 59-63.
3. Tagantsev, A. K., Landau Expansion for Ferroelectrics: Which Variable to Use? *Ferroelectrics* 2008, 375, 19-27.
4. Haun, M. J.; Furman, E.; Jang, S. J.; McKinstry, H. A.; Cross, L. E., Thermodynamic theory of PbTiO 3. *Journal of Applied Physics* 1987, 62, 3331-8.
5. Li, Y. L.; Cross, L. E.; Chen, L. Q., A phenomenological thermodynamic potential for BaTiO3 single crystals. *Journal of Applied Physics* 2005, 98.
6. Wang, Y. L.; Tagantsev, A. K.; Damjanovic, D.; Setter, N.; Yarmarkin, V. K.; Sokolov, A. I., Anharmonicity of BaTiO3 single crystals. *Physical Review B* 2006, 73.
7. Landau, L. D.; Lifshitz, E. M.; Pitaevskii, L. P., Electrodynamics of Continuous Media. *Oxford University Press* 1984.
8. Kretschmer, R.; Binder, K., Surface effects on phase transitions in ferroelectrics and dipolar magnets. *Physical Review B* 1979, 20, 1065-1076.
9. Sluka, T.; Tagantsev, A. K.; Damjanovic, D.; Gureev, M.; Setter, N., Enhanced electromechanical response of ferroelectrics due to charged domain walls. *Nat Commun* 2012, 3, 748.
10. Hlinka, J.; Márton, P., Phenomenological model of a 90° domain wall in BaTiO_{3}-type ferroelectrics. *Physical Review B* 2006, 74, 104104.
11. Zhong, W. L.; Wang, Y. G.; Zhang, P. L.; Qu, B. D., Phenomenological study of the size effect on phase transitions in ferroelectric particles. *Physical Review B* 1994, 50, 698-703.
12. a_1=4.124(T-388)$\times10^5$, a_{11}=-2.097$\times10^8$, a_{12}=7.974$\times10^8$, a_{111}=1.294$\times10^9$, a_{112}=-1.950$\times10^9$, a_{123}=-2.500$\times10^9$, a_{1111}=3.863$\times10^{10}$, a_{1112}=2.529$\times10^{10}$, a_{1122}=1.637$\times10^{10}$, a_{1123}=1.367$\times10^{10}$, Q_{11}=0.1104 , Q_{12}=-0.0452 , Q_{44}=0.0289 , s_{11}=6.31$\times10^{-12}$, s_{12}=-4.11$\times10^{-12}$, s_{44}=18.42$\times10^{-12}$, G_{11}=51$\times10^{-11}$, G_{12}=0 , $G_{44}=G'_{44}=1\times10^{-11}$ and $\varepsilon_b \approx 4.425\times10^{-10}$ (SI units and T in K).
